# Extrusion-Based Printing of Myoblast-Loaded Fibrin Microthreads to Induce Myogenesis

**DOI:** 10.3390/jfb16010021

**Published:** 2025-01-10

**Authors:** Hanson S. Lee, Bryanna L. Samolyk, George D. Pins

**Affiliations:** Department of Biomedical Engineering, Worcester Polytechnic Institute, Worcester, MA 01609, USA; hlee3@wpi.edu (H.S.L.); bsamolyk@wpi.edu (B.L.S.)

**Keywords:** fibrin microthreads, bioprinting, cell alignment, myotube formation, volumetric muscle loss, skeletal muscle, biomaterials, tissue engineering, tensile properties

## Abstract

Large skeletal muscle injuries such as volumetric muscle loss (VML) disrupt native tissue structures, including biophysical and biochemical signaling cues that promote the regeneration of functional skeletal muscle. Various biofabrication strategies have been developed to create engineered skeletal muscle constructs that mimic native matrix and cellular microenvironments to enhance muscle regeneration; however, there remains a need to create scalable engineered tissues that provide mechanical stability as well as structural and spatiotemporal signaling cues to promote cell-mediated regeneration of contractile skeletal muscle. We describe a novel strategy for bioprinting multifunctional myoblast-loaded fibrin microthreads (myothreads) that recapitulate the cellular microniches to drive myogenesis and aligned myotube formation. We characterized myoblast alignment, myotube formation, and tensile properties of myothreads as a function of cell-loading density and culture time. We showed that increasing myoblast loading densities enhances myotube formation. Additionally, alignment analyses indicate that the bioprinting process confers myoblast alignment in the constructs. Finally, tensile characterizations suggest that myothreads possess the structural stability to serve as a potential platform for developing scalable muscle scaffolds. We anticipate that our myothread biofabrication approach will enable us to strategically investigate biophysical and biochemical signaling cues and cellular mechanisms that enhance functional skeletal muscle regeneration for the treatment of VML.

## 1. Introduction

Each year 5.7 million Americans suffer from traumatic injury due to car accidents, combat, or tumor ablation, costing an estimated USD 20 billion [1,2,3,4]. These injuries result in significant damage to soft tissues of the limbs, neck, and face, including underlying muscle tissue. While skeletal muscle exhibits tremendous potential to repair itself after minor injuries, severe muscle trauma can lead to volumetric muscle loss (VML), exceeding the endogenous regenerative capacity of the tissue. These VML injuries disrupt native tissue structures and mechanical supports, as well as the biochemical and biophysical signaling cues from growth factor (GF) reservoirs and extracellular matrix (ECM) receptors in the satellite cell microniches [5]. Loss of these signaling cues limits the ability of the damaged tissue to direct aligned myogenesis, tissue vascularization, reinnervation and functional muscle regeneration. The standard of care for treating craniofacial VML injuries consists of free muscle flap transfers (FFTs), which commonly result in donor site morbidity, subpar tissue innervation and scar tissue deposition, leading to poor functional recovery [5,6,7,8,9,10,11,12,13,14,15]. As such, there remains a significant need to develop new therapies that promote the functional restoration of contractile muscle tissue to treat VML injuries. Specifically, it is critical to develop tissue scaffolds for skeletal muscle repair that precisely recapitulate the biophysical and biochemical cues in cellular microniches that instruct cell-mediated myogenesis and the regeneration of functional tissue [5,6,7,8,9,10,11,12,13,14,15].

Tissue engineering technologies offer great promise for promoting muscle regeneration. Numerous three-dimensional (3D) scaffolds with biophysical and biochemical cues that mimic native satellite cell microniches have been investigated to deliver sustained amounts of GFs, regenerative biomolecules, genes, and cellular therapies in a variety of injury models [5,6,7,12,14,16,17,18,19,20,21,22,23,24,25,26,27,28,29,30,31,32,33,34,35,36]. These approaches are generally classified as acellular scaffolds composed of synthetic or biological cell-instructive materials, or cellular scaffolds that combine implantable materials with stem cells, muscle progenitor cells (MPCs) or myoblasts [37,38,39]. Acellular scaffolds with a variety of compositions and architectures have demonstrated limited success for repairing muscle injuries in both preclinical and clinical studies [8,13,38,39,40]. These therapies work by delivering sustained doses of biochemical cues such as GFs to stimulate the recruitment, proliferation and differentiation of local progenitor cells at the injury site. However, more complete functional regeneration requires precise control of the spatiotemporal distribution of critical GFs over a physiologically relevant period of time [5,6,8,9,12,41,42]. In contrast, cellular scaffolds enable the delivery of progenitor cells that facilitate exogenous regenerative responses, augmenting endogenous tissue repair [6,7,8,10,43,44,45,46]. Despite regaining modest functional regeneration, many of these injuries still contain significant scar tissue, misaligned myofibers, and limited cellular infiltration into the wound site [6,7,8,10,36].

Numerous biofabrication strategies have been investigated to develop skeletal muscle constructs that replicate the biophysical and biochemical cues of the native matrix and incorporate cells to enhance functional tissue recovery and reduce fibrosis [5,7,10,12,47]. Advanced manufacturing techniques such as electrospinning and 3D bioprinting commonly use natural biopolymers including decellularized ECM, collagen, fibrin, alginate, laminin, silk fibroin, hyaluronic acid (HA), chitosan, keratin, and gelatin to create structures such as sheets, fibers, microparticles, hydrogels and composite materials to enhance the signaling cues in regenerative cellular microniches [21,23,30,33,45,48,49,50,51,52,53,54,55,56,57,58,59,60,61,62,63,64,65,66,67,68,69,70]. To improve functional recovery, these scaffolds are often loaded with myogenic cells such as MPCs, skeletal muscle stem cells (satellite cells) or induced pluripotent stem cells (iPSCs) to direct mechanisms that increase the rate of myogenesis and contractile skeletal muscle regeneration [14,18,28,45,47,48,56,64,71]. Despite some success of these engineered tissues, there remains a need to create scalable tissue constructs that provide robust mechanical stability, cell alignment, and spatiotemporal control over the presentation of bioactive signaling cues to promote the rapid regeneration of contractile skeletal muscle [5,10].

Towards this goal, our laboratory developed a facile strategy for fabricating multifunctional fibrin-based myoblast-loaded microthread constructs (myothreads) that recapitulate native cellular microniches to direct cell-mediated myogenesis. Previously, we described methods for using fibrin, a native pro-regenerative biopolymer, to fabricate microthreads with fiber-like architecture that provides structural and biochemical signaling cues to promote skeletal muscle regeneration [72,73,74]. When bundles of fibrin microthreads were loaded with hepatocyte growth factor (HGF) or seeded with skeletal muscle cells and implanted into a tibialis anterior (TA) model of a VML defect, they promoted functional recovery of approximately 80% of native tissue force [18,40]. Here, we describe an approach for creating engineered tissue constructs that further mimic native tissue, by printing myothreads, systematically varying the scaffold design parameters and evaluating the effect of myoblast densities (1–4 × 10^6^ myoblasts/mL) on scaffold morphology, following 1–2 weeks of tissue culture. We hypothesize that increasing myoblast densities will enhance nuclear alignment, myogenesis, and the rate of myotube formation, as well as the mechanical stability of the tissue constructs. The results of these studies suggest that extruded fibrin-based myothreads offers a promising approach for creating hierarchically ordered engineered skeletal muscle constructs, composed of structural elements comparable to native tissue.

## 2. Materials and Methods

### 2.1. C2C12 Myoblast Culture

Immortalized mouse myoblasts (C2C12, ATCC CRL-1772) were cultured in C2C12 complete medium consisting of 1:1 (*v*/*v*) ratio of high glucose, no glutamine, Dulbecco’s Modified Eagle Medium (DMEM, Gibco, Thermo Fisher Scientific, Waltham, MA, USA, Cat No. 11960-044) and Ham’s F12 medium (Gibco, Thermo Fisher Scientific, Waltham, MA, Cat No. 11765054), 10% fetal bovine serum (FBS, HyClone, Logan, UT, USA, Cat No. SH30071.03), 50 IU/mL penicillin-streptomycin (P/S, Corning, Corning, NY, USA, Cat No. 30-002) and 1.25 µg/mL amphotericin-B (Amp B, Sigma Aldrich, Burlington, MA, USA, Cat No. A2942). Cells were incubated in 5% CO_2_ at 37 °C and maintained below 70% confluency using standard cell culture techniques. Each passage was carried out with 0.25% trypsin-EDTA (Corning, Corning, NY, USA, Cat No. 25-053-Cl). For all experiments, passage numbers were between 8 and 10.

### 2.2. Biofabrication of Fibrin Scaffolds and Myothreads

**Figure 1:**
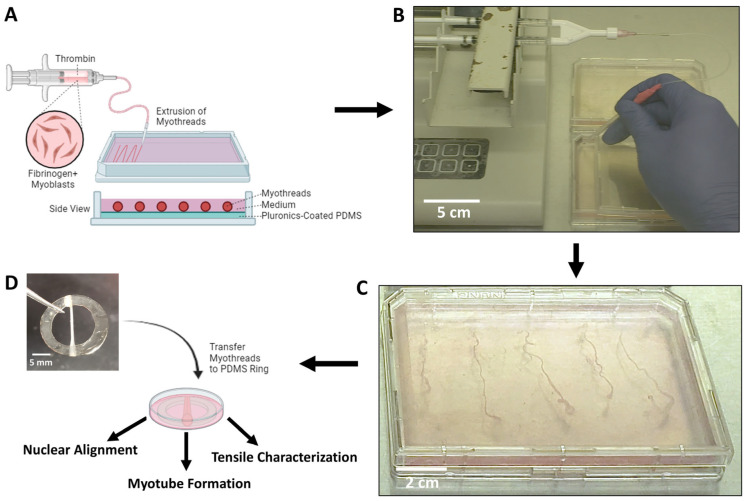
Schematic illustration detailing the stepwise process for biofabrication of myothreads. (**A**) Cartoon illustrating the bioprinting setup with labelled components. (**B**) Photos showing the myofiber coextrusion process and (**C**) bundled myothreads in extrusion bath. (**D**) Wrapped myothread on PDMS ring for culture and characterization.

Bovine fibrinogen (MP Biomedical, Irvine, CA, USA, Cat No. 08820226) was reconstituted at 10 mg/mL in HEPES-buffered saline (HBS) ((N-[2-Hydroxyethyl] piperazine-N¢-[2-ethanesulfonic acid], 20 mM HEPES (Sigma Aldrich, Burlington, MA, USA, Cat No. H3375), 0.9% NaCl (Sigma Aldrich, Burlington, MA, USA, Cat No. 9888), pH 7.4,), prefiltered with low-protein binding 0.44 µm filter units (EMD Millipore, Burlington, MA, USA, Cat No. SLHVM33RS) and stored at −20 °C prior to use. Aliquots of 40 U/mL thrombin from bovine plasma (Sigma Aldrich, Burlington, MA, Cat No. T4648) was prepared in HBS, prefiltered with 0.44 µm filter, and stored at −20 °C until use. The myothread extrusion kit consisting of polyethylene tubing with 0.86 mm inner diameter (BD, Sparks, MD, USA; Cat No. 14-170-12N) and Y-connector blending applicator (Micromedic Inc., St. Paul, MN, USA; Cat No. SA-3670) was sterilized for 2 h in 70% ethanol incubation under UV light, followed by three rinses with sterile water, and dried overnight. Prior to extrusion, autoclaved PDMS plates (DOW Inc., Midland, MI, USA, Cat No. 1317318) and extrusion kits were incubated with sterile 1% Pluronics (Sigma Aldrich, Burlington, MA, USA, Cat No. P2443) dissolved in deionized water (di-H_2_O) for at least 1 h. Initial densities of C2C12 myoblasts (1 × 10^6^ cells/mL, 2 × 10^6^ cells/mL, or 4 × 10^6^ cells/mL) were pre-incubated in C2C12 complete medium at 37 °C in 15 mL conical tube for 2 h, resuspended and centrifuged at 125× *g* for 5 min and resuspended in 10 mg/mL of fibrinogen [48,75,76]. Aliquots of 40 U/mL thrombin were diluted in 40 mM CaCl_2_ to achieve final working concentrations of 8 U/mL. Fibrinogen solutions (10 mg/mL) without C2C12 myoblasts were used as controls. Individual syringes with equal volumes (1 mL) of fibrinogen and thrombin solutions were inserted into the Y-connector kit and extruded at a flow rate of 0.225 mL/min via a dual syringe pump (KD Scientific, Holliston, MA, USA) onto sterile PDMS sheets into a pre-warmed 37 °C myothread extrusion buffer (MEB) composed of 50% (*v*/*v*) DMEM (Dulbecco’s Modified Eagle Medium, Thermo Scientific, Waltham, MA, USA, Cat No. 12100046), 25% (*v*/*v*) DPBS (Dulbecco’s Phosphate-Buffer Saline, Corning, Corning, NY, USA, Cat No. 20-031-CV), and 25% (*v*/*v*) deionized water, pH 7.0 (Figure 1). Temporal control of the extrusion rate of individual myothreads ensured consistent material was dispensed for each scaffold. After the myothreads were incubated for 15 min, 4 extruded threads were combined, stretched 10% of their original length measured with a calibrated scale, and loaded onto PDMS rings (Figure 1). For control studies, myoblast-loaded fibrin gels were constructed on sterile glass 18 mm × 18 mm cover slips (VWR, Radnor, PA, USA, Cat No. 48366 045) by mixing equal volumes of 10 µL of fibrinogen (10 mg/mL) containing aforementioned myoblast densities with 10 µLs of thrombin composition used for myothread construction onto 4 corners and the center of the cover slip. After 15 min of fibrin polymerization, the gels were fixed and Hoechst-stained for nuclear imaging using the manufacturer’s protocol. Myothreads were cultured in complete medium with 20 µg/mL aprotinin (Sigma Aldrich, Burlington, MA, USA, Cat No. A1153) for 3 days. For differentiation, myothreads were transferred to differentiation medium containing 1:1 DMEM/F-12 supplemented with 2% horse serum (HyClone, Logan, UT, USA, Cat No. SH30074.03), 1% Insulin-Transferrin-Selenium (ITS; Thermo Fisher Scientific, Waltham, MA, USA, Cat No. 45001-090), 50 IU/mL P/S, 1.25 µg/mL Amp B, and 20 µg/mL aprotinin and incubated in differentiation medium for 6 days, with medium changes every 2 days.

### 2.3. Assessment of Myoblast Viability in Myothread

Myoblast viability in myothreads was evaluated on Day 1 and Day 9 of culture using a LIVE/DEAD stain (Sigma Aldrich, Burlington, MA, Cat No. L3224). Myothreads were stained with a DPBS solution (Corning, Corning, NY, Cat No. 20-030-CV) containing 1:1000 Calcein-AM (C-AM), 1:250 Ethidium Homodimer-1 (EtH-1), and 1:2000 Hoechst (Thermo Fisher Scientific, Waltham, MA, Cat No. H3570) for 30 min at room temperature. Myothreads were then transferred into DPBS and imaged on Leica SP5 Point Scanning Confocal Microscope (Leica Microsystems, Deerfield, IL, USA). Images formatted as 1024 × 1024 pixels were collected at 0.065/s frame rate with 4 µm/step. Images were processed using ImageJ software v1.73 (Bethesda, Maryland, NIH) [77]. Viability percentages were quantified by dividing the difference between total cells stained by Hoechst and cells stained by EtH-1 over the total cells stained by Hoechst.

### 2.4. Quantification of Myoblast Alignment and Myotube Formation

Myothread morphology was characterized by analyzing confocal images of nuclear alignment and myotube formation (MHC^+^ nuclei %, myotube architecture, length, and area) as a function of myoblast-seeding density. Images were formatted as 1024 × 1024 pixels and were collected at 0.065/s frame rate with 2.5 µm/step. Myothreads were imaged along the long axes of the threads and individual Z-slices from myothreads were captured in three discrete sections of each sample, background corrected and thresholded with Image J v.1.73. Myotube formation was analyzed by calculating the percentage of cells expressing markers for both nuclei and myosin heavy chain (MHC), using CellProfiler software v4.2.1 (Broad Institute, Cambridge, MA) [78]. To characterize myotube dimensions, MHC-stained cells containing ≥ 3 nuclei were analyzed for myotube area, length, and diameter using ImageJ. Myoblast alignment was calculated by measuring the angles between the major axes of Hoechst-stained nuclei and the long axes of myothreads, using ImageJ, at the initial bioprinting time (day 0) and after 9 days of cell culture. Confocal images of myothreads were processed with a background removal tool and local Bernsen threshold analyses to declump and segment individual nuclei for characterization using ImageJ. Nuclear alignment angles were binned in 15° increments and histograms displayed total nuclear alignment distribution. Resulting orientation distribution curves were adjusted to 0° alignment, corresponding to the long axis of the myothreads. Nuclei aligned within ± 15° were calculated as the percentage of aligned nuclei divided by the total nuclei for *n* = 4 samples. Clumped nuclei were disqualified from the analysis.

### 2.5. Characterization of Myothread Morphology

To characterize myoblast morphology, myothreads were washed in DPBS with calcium and magnesium (Corning, Corning, NY, USA, Cat No. 20-030-CV) (2 × 5 min) and fixed in 4% paraformaldehyde PBS solution (Thermo Fisher Scientific, Waltham, MA, USA, Cat No. T353) for 30 min. After PBS rinses (3 × 5 min), samples were incubated in 1% Sudan Black Solution (Sigma Aldrich, Burlington, MA, Cat No. 199664) in 70% ethanol for 24 h at 4 °C on a shaker plate at 150 rpm (VWR, DS-500, Radnor, PA, USA, Cat No. 57018-754). Unbound Sudan Black Solution was washed with PBS rinses (3 × 5 min) [79,80]. Samples were permeabilized in 0.2% Triton-X PBS solution (Sigma Aldrich, Burlington, MA, Cat No. X100) for 30 min and blocked with 10% Goat Serum/PBS (GS, Abcam, Waltham, MA, USA, Cat No. AB7481) for 1 h [48]. Primary antibodies targeting Myosin Heavy Chain (1:40; MHC; Hybridoma Bank, Iowa City, IA, USA, Cat No. MF-20) in 5% GS was loaded onto the samples for 24 h at 4 °C, on a shaker plate. The negative controls did not include the primary antibody. The samples were rinsed in 0.1% Tween-20/PBS (PBS-T; Thermo Fisher Scientific, Waltham, MA, USA, Cat No. BP337, 3 × 5 min) and incubated with FITC-conjugated goat anti-mouse antibodies (1:2000; Thermo Fisher Scientific, Waltham, MA, USA, Cat No. A11001) in 5% GS overnight, on a shaker plate. After removing unbound antibodies with PBS rinses (3 × 5 min), F-actin was stained with Phalloidin 568 (1:400, Thermo Fisher Scientific, Waltham, MA, USA, Cat No. A12380) in 1% BSA (Sigma Aldrich, Waltham, MA, Cat No. A2153) for 1 h at room temperature (RT). Following PBS rinses (3 × 5 min), cell nuclei were stained with Hoechst/PBS solution (1:2000) at 4C, overnight, on a shaker plate. Samples were rinsed in PBS (3 × 5 min), mounted on glass cover slides with mounting medium (Thermo Fisher Scientific, Waltham, MA, USA, Cat No. 8310-4) and imaged on a Leica SP5 Point Scanning Confocal Microscope (Leica Microsystems, Deerfield, IL, USA). Images formatted as 1024 × 1024 pixels were collected at 0.065/s frame rate with 2.5 µm/step.

### 2.6. Mechanical Characterization of Myoblast-Loaded Fibrin Microthreads

Mechanical properties of cell-loaded fibrin myothreads were analyzed as a function of myoblast densities and culture times, using methods described previously [72,81]. Immediately after culture, myothreads were loaded onto parchment frames (Katbite, FR) with precut windows of 10 mm, which defined the loading region as well as the gauge length of the samples (10.0 mm). Myothreads were adhered onto the frames with moisture-activated cyanoacrylate Super Glue adhesive (The Gorilla Glue Company, Cincinnati, OH, USA). After briefly drying, the samples were rinsed and hydrated in PBS. Diameters from three different thread regions were measured using a calibrated reticule with a 10× objective on a Nikon Eclipse E600 inverted microscope (Nikon, Melville, NY, USA). To define the cross-sectional area, microthreads were assumed to be cylinders, and three diameter measurements were calculated along the length of each myothread, and averaged [48,72,73,82]. Frames with myothreads were submerged in a PBS bath and loaded into the clamps of an ElectroPuls E1000 uniaxial testing machine (Instron, Norwood, MA, USA) fixtured with a 1 N load cell. Once secured, the two edges of parchment frames were cut, and myothreads were uniaxially loaded until failure at a 50% strain rate (5 mm/min). Force and displacement were recorded continuously at a frequency of 10 Hz. Engineering stress was calculated by dividing the recorded force by the initial cross-sectional area. The ultimate tensile strength (UTS), linear elastic modulus (calculated at a range between 0 and 20% total length stretched) (E), and strain at failure (SAF), as well as load at failure for each sample were determined using a custom MATLAB R2024a (Mathworks, Natick, MA, USA) script.

### 2.7. Statistical Analysis

Statistical analyses were performed using GraphPad Prism 10 software (GraphPad Software, Boston, MA, USA). Studies were conducted at least 3 times with *n* ≥ 3 samples per condition. A Shapiro–Wilk test evaluated the normality of all datasets [83]. For non-normal datasets, outliers were removed using a Grubbs test, and normality was re-evaluated [84]. For normal data, the appropriate analysis of variance (ANOVA) (*p* < 0.05) followed by Tukey’s post hoc analysis was used. For non-normal data, Kruskal–Wallis (*p* < 0.05) and Dunn’s multiple comparison was used. All values were reported as mean ± standard deviation (SD).

## 3. Results

### 3.1. Myothread Extrusion Maintains Myoblast Viability and Promotes Alignment

**Figure 2:**
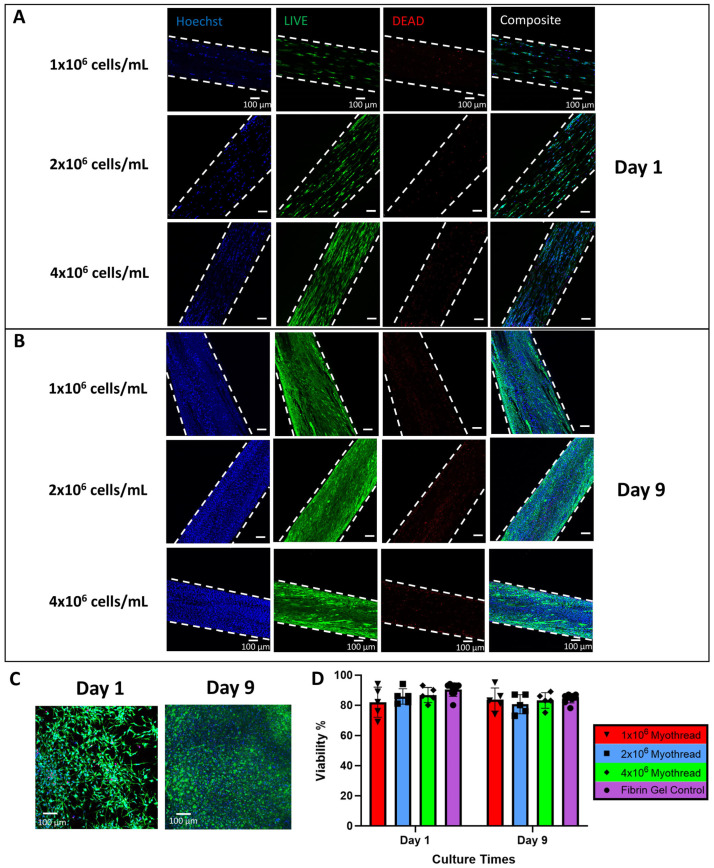
Viability Assessment of Myothreads. (**A**) Representative LIVE/DEAD confocal images of myothreads stained after 1 day of culture and (**B**) 9 days of culture. Hoechst stains for total cell count. Scale bars are 100 µm. (**C**) Representative of fibrin gel (4 × 10^6^) consists of all myoblast conditions as a control (*n* = 9). (**D**) Plots show the viability percentage for each myoblast density from different culture times (*n* = 5). Mean ± SD.

**Figure 3:**
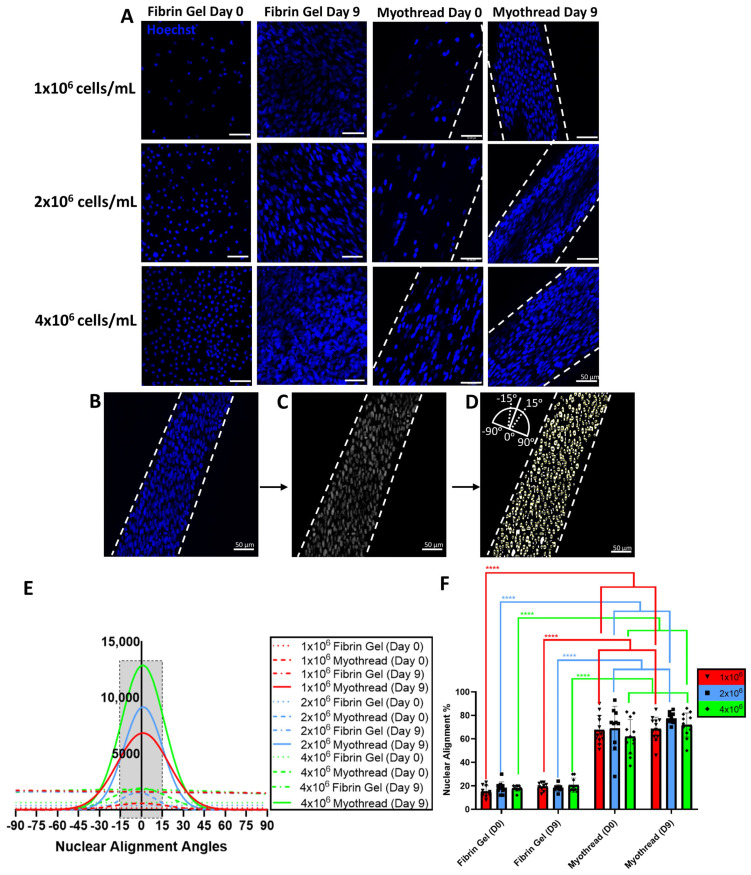
Nuclear alignment analyses of myothreads. (**A**) Representative confocal images of nuclear stains for myoblast configurations in varied fibrin scaffold and culture conditions. Scale bars are 50 µm. Schematic images illustrate the nuclear alignment strategy, including (**B**) the white dashed line defining the offset angle for the imaged thread, (**C**) a gray-scale image integrating contrast and background subtraction to declump the nuclei with ImageJ and (**D**) a particle analysis (ImageJ) providing angles that were subtracted from the measured offset angle, shown in the inset. (**E**) Plots showing nuclear alignment distribution for each fibrin scaffold configuration, myoblast density, and culture condition. Gray box highlights alignment region of ±15 degrees. (**F**) Quantification of nuclei percentage aligned within 15 degrees (N = 3, *n* = 4). Mean ± SD, **** indicates *p* < 0.0001.

To characterize the myoblast alignment in myothreads, scaffolds were fabricated with varying densities of C2C12 myoblasts and cultured for 9 days to promote myotube formation. Confocal images of myothreads were analyzed to assess viability and nuclear orientation of the cells as a function of cell-loading density and time (Figure 2A–C and Figure 3A–D). All myothreads and fibrin gel controls exhibited at least 80% viability for different myoblast densities and culture time points (Figure 2D). All myothreads exhibited mean nuclear alignment values of at least 60%, independent of cell culture time or scaffold seeding density (Figure 3E,F). Myothreads exhibited the initial nuclear alignment values of 67.9 ± 15.8%, 69.1 ± 18.7%, or 61.6 ± 14.7% for scaffolds fabricated with 1 × 10^6^, 2 × 10^6^, or 4 × 10^6^ myoblasts/mL, respectively. Myothreads cultured for 9 days exhibited similar nuclear alignment values, 68.6 ± 10.0%, 77.5 ± 4.3%, or 71.7 ± 10.2% for scaffolds with 1 × 10^6^, 2 × 10^6^, or 4 × 10^6^ myoblasts/mL, respectively (Figure 3E,F). There were no statistically significant differences in myoblast alignment as a function of initial cell-seeding density or culture time. In contrast, myoblast-seeded fibrin gels (negative controls) had nuclear alignment values of 15.2 ± 4.9%, 18.0 ± 5.2%, or 17.8 ± 2.3%, for gels fabricated with 1 × 10^6^, 2 × 10^6^, and 4 × 10^6^ myoblasts/mL at Day 0 and 19.3 ± 3.6%, 18.0 ± 2.7%, and 20.5 ± 6.1% at Day 9 (Figure 3A,E). All 3D-printed myothreads exhibited statistically significantly higher nuclear alignment values than the C2C12-seeded fibrin gels at all densities.

### 3.2. Initial Myoblast Quantities Modulate Myotube Formation in Fibrin Microthreads

**Figure 4:**
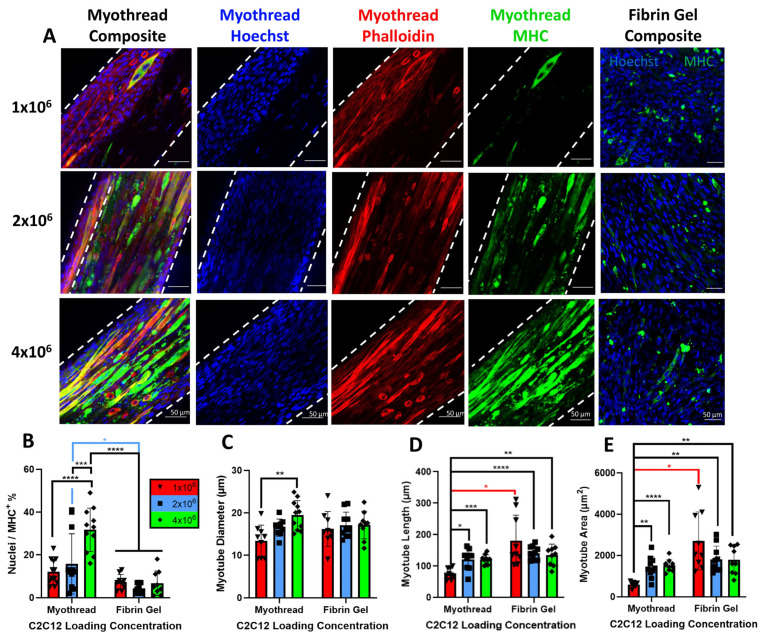
Characterization of myotube formation in myoblast-loaded fibrin microthreads (myothreads). Myotube formation in myoblast-loaded fibrin microthreads and fibrin gel cultured for 9 days. (**A**) Representative immunofluorescence showing nuclear (Hoechst), f-actin (phalloidin) and myosin heavy chain (MHC) staining of myothreads at day 9 of culture and MHC and Hoechst images of myoblast-loaded fibrin gels as controls at day 9 of culture. Scale bars are 50 µm. Plots showing (**B**) percentage of nuclei/MHC positive stained cells. Myotubes (selected as z-projected cells stained with MHC and at least three nuclei) were analyzed by (**C**) myotube diameter, (**D**) myotube length, and (**E**) myotube area as a function of C2C12 loading concentrations (cells/mL) values (N = 3, *n* = 4). Mean ± SD, * indicates *p* < 0.05, ** indicates *p* < 0.01, *** indicates *p* < 0.001, **** *p* < 0.0001.

To evaluate myogenesis as a function of cell-loading densities, we constructed myothreads with varied myoblast densities and we analyzed myotube morphologies after culturing cell-seeded threads for 9 days. These analyses showed that the percentage of nuclei/MHC^+^ cells significantly increased with myoblast extrusion quantity. Threads extruded with 4 × 10^6^ cells/mL exhibited significantly more nuclei/MHC^+^ cells (32.8 ± 10.2%) than threads extruded with 1 × 10^6^ cells/mL (11.9 ± 5.8%; *p* < 0.0001) or 2 × 10^6^ cells/mL (15.9 ± 14.0%, *p* < 0.001), as well as fibrin gel controls with values of 7.4 ± 3.6%, 4.4 ± 2.1%, and 6.8 ± 5.9% for 1 × 10^6^, 2 × 10^6^, and 4 × 10^6^ myoblasts/mL, respectively (*p* < 0.0001) (Figure 4B). Myotube morphologies formed in myothreads were further characterized by the diameters, lengths, and areas of the discrete structures within the myothreads. Myotube diameters were 13.4 ± 3.7 µm, 15.8 ± 5.3 µm, or 19.5 ± 3.5 µm for scaffolds seeded with 1 × 10^6^ cells/mL, 2 × 10^6^ cells/mL, or 4 × 10^6^ cells/mL, respectively. Myothreads fabricated with 4 × 10^6^ cells/mL exhibited a statistically significant increase in myotube diameter, relative to myothreads fabricated with 1 × 10^6^ cells/mL (Figure 4C). Threads extruded with 2 × 10^6^ and 4 × 10^6^ cells/mL exhibited significantly higher average myotube lengths (121.6 ± 33.2 and 120.7 ± 14.9 µm, respectively) and areas (1427.4 ± 514.6 µm^2^ and 1528.1 ± 277.5 µm^2^, respectively) than threads extruded with 1 × 10^6^ cells/mL, myotube lengths (76.0 ± 17.0 µm), and areas (631.9 ± 172.3 µm^2^). While myothreads with 2 × 10^6^ and 4 × 10^6^ had no statistical difference in myotube length and area with the fibrin gel controls, myothreads extruded with 1 × 10^6^ cells/mL exhibited statistically significant differences in myotube length and area relative to the myoblast-loaded fibrin gel controls (Figure 4D,E).

### 3.3. Myoblasts Modify Final Tensile Properties of Myothread

**Figure 5:**
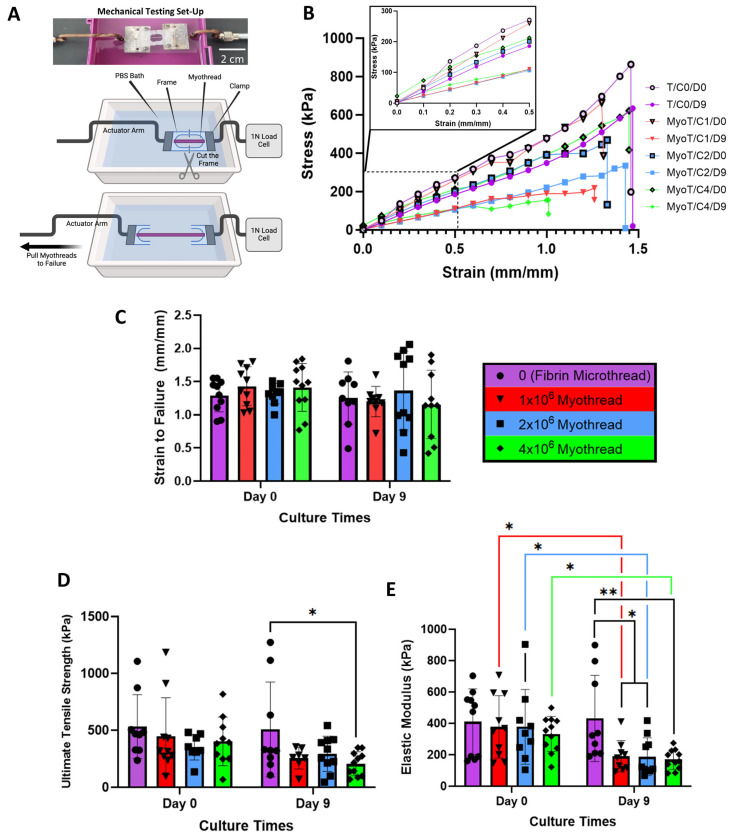
Tensile mechanical characterization of myothreads. (**A**) Photo (top) and cartoon configuration (bottom) of fibrin myothreads mounted on vellum frames in buffer bath for uniaxial tensile testing. (**B**) Representative stress/strain curves of myothreads with varied myoblast densities and culture time points. Plots showing analyses of (**C**) strains to failure, (**D**) ultimate tensile strengths, (**E**) elastic moduli at 20% strain (N = 3, *n* = 3–4), mean ± SD. * indicates *p* < 0.05, ** indicates *p* < 0.01. Legend nomenclature denotes acellular (T), or cell-loaded (MyoT) fibrin myothreads loaded with 0 (C0), 1 × 10^6^ (C1), 2 × 10^6^ (C2), or 4 × 10^6^ (C4) myoblasts/mL and culture for 0 (D0) or 9 (D9) days.

To evaluate the effect of myoblast densities on structural and mechanical properties of myothreads, scaffolds were analyzed in uniaxial tension under PBS-buffered conditions and loaded to failure (Figure 5A). Representative stress–strain curves, thread dimensions and tensile characteristics are summarized in Figure 5B and Table 1. Scaffold diameters (387 ± 83 to 469 ± 98 mm) and strains at failure (SAF) (1.14 ± 0.52 to 1.46 ± 0.50) did not vary significantly as a function of cell-loading density or culture time (Figure 5C). Cultured myothreads exhibited decreased ultimate tensile strengths (UTSs), and elastic moduli (E). All cell-loaded myothreads exhibited a significant decrease in modulus after 9 days of culture, relative to myothreads tested immediately after bioprinting. Myothreads loaded with 2 × 10^6^ or 4 × 10^6^ myoblasts/mL exhibited a statistically significant decrease in scaffold modulus relative to acellular fibrin microthread controls after 9 days of cell culture (Figure 5E). Additionally, myothreads loaded with 4 × 10^6^ myoblasts/mL exhibited a statistically significant decrease in tensile strength relative to acellular fibrin microthread controls after 9 days of cell culture (Figure 5D).

## 4. Discussion

The long-term goal of this project is to develop an implantable scaffold that recapitulates the native tissue biochemical and biophysical signaling cues that direct cell-mediated myogenesis, angiogenesis and reinnervation, and that increase the functional contractile properties of engineered skeletal muscle for the treatment of VML injuries [5,6,7,12,85]. Tissue-engineered skeletal muscle constructs combine myogenic cells and bioactive scaffolds to drive tissue regeneration at the wound site. However, the utility of these strategies remains limited by insufficient maturation of the skeletal muscle tissue and incomplete functional recovery [5,12,18,50,86,87]. As such, there remains a significant need to create implantable scaffolds that enhance aligned skeletal muscle maturation, tissue vascularization, and innervation, as well as complete functional recovery [5,7,10,11,12,36]. Towards this objective, our laboratory developed a novel fibrin-based scaffold bioprinting system that mimics native skeletal muscle architecture with thread-like geometry, and regenerative signaling cues that promote myogenic outcomes for myoblast-loaded scaffolds. To our knowledge, this is the first study to systematically investigate the myogenic potential of myoblast-loaded fibrin microthreads (myothreads). Our findings suggest that these myothreads provide structural signaling cues that increase myogenesis in a dose-dependent manner. These findings also suggest that extruded myothreads may serve as a platform technology to systemically probe biological mechanisms and multicellular signaling interactions that enhance skeletal muscle maturation and promote tissue vascularization and innervation for future studies.

In this study, we observed that the rate of myotube formation in the fibrin myothreads increased as a function of the initial myoblast-loading density used during the bioprinting process. We attribute this outcome to two findings that we noted during the fabrication of the cell-loaded scaffolds. First, we observed that the myothread extrusion process maintained myoblast viability and significantly enhanced myoblast alignment within the scaffolds, independent of cell-loading density or cell culture time. This finding is consistent with previous studies showing that the extrusion of cell-loaded engineered muscle scaffolds with anisotropic scaffold architectures and shear forces from extrusion promote guided cell alignment and maintain cell viability [53,64,72,74,82,88,89,90,91,92]. Second, we observed a dose-dependent increase in the percentage of nuclei/MHC^+^ cells and an increase in the dimensions of the myotubes within the scaffolds. Interestingly, while the 2 × 10^6^ and 4 × 10^6^ cell/mL loaded threads had a statistically higher MHC^+^ nuclei percentage than the fibrin gel controls, they did not exhibit difference in myotube formation morphology from the myoblast-loaded fibrin gel. Together, these findings are consistent with previous studies showing that increased myogenic cell densities and aligned myoblasts enhance myotube formation by promoting the reorganization of adjacent surface adhesion markers that signal myoblast fusion and multi-nucleated myotube formation [93,94,95]. Similarly, Cheng et al. demonstrated that increasing primary human myoblast (HSkM) loading densities from 5 × 10^6^ to 1.5 × 10^7^ HSkM/mL enhanced myotube formation and contraction force output after 14 days of culture [96]. Previous studies also demonstrated that enhanced nuclear alignment during the biofabrication of engineered muscle scaffolds correlated with enhanced functional recovery of skeletal muscle tissue [92,97,98,99]. Additionally, Corona et al. demonstrated that seeding muscle progenitor cells (MPCs) on two sides of bladder acellular matrix (BAM) scaffolds increased myosin expression and multi-nucleated cell formation relative to single-sided seeding of BAM scaffolds. Lewis rats implanted with BAM scaffolds that were seeded on two sides exhibited higher isometric force output than rats receiving the single-side seeded BAMs (10^6^ MPC/cm^2^) [100]. Kim et al. characterized fibrin constructs that were loaded with a range of human muscle progenitor cells (hMPC; 1–5 × 10^7^ cells/mL), and determined that 3 × 10^7^ and 5 × 10^7^ cells/mL had the highest quantity of myotubes in vitro and the greatest area of MHC^+^ myofibers quantities, both in vitro and in vivo [71]. Together, these findings suggest that in vitro analyses of myoblast alignment and myotube formation in implantable scaffolds are reliable predicators of enhanced functional recovery after implantation. Thus, as part of a future investigation, we will evaluate whether myothreads can support higher cellular densities and serve as scalable multifunctional tissue elements that can be precisely engineered in large-scale muscle constructs to promote functional tissue regeneration.

Mechanical characterizations of the cell-loaded myothreads developed in this study highlight their utility as fibrin-based myogenic cell-delivery scaffolds. Interestingly, we observed that there were no significant differences in the diameters, loads and strains at failure or the ultimate tensile strengths of cell-loaded myothreads as a function of culture time or cell-loading density. Furthermore, the loads and strains to failure, as well as ultimate tensile strengths of these myothreads, were not significantly different to the acellular, control fibrin myothreads, except for myothreads loaded with 4 × 10^6^ cells/mL that were cultured for 9 days. These findings suggest that myothread culture may not significantly affect the functional or handling characteristics of the constructs, but the statistical difference between myothreads cultured with 4 × 10^6^ cells/mL and acellular fibrin microthreads suggest that quantitative histomorphometric investigations to evaluate the matrix deposition within the myothreads should be pursued [74]. Further, cultured myothreads exhibited decreased elastic moduli values as a function of cell culture time. These cell-loaded myothreads exhibited elastic moduli that were comparable to previously published elastic-moduli values [62,64,101,102,103,104]. In one study, Fan et al. showed that bio-printed 0.6 mm fibrin-bundle scaffolds cultured for 7 days with 10^7^ C2C12 myoblasts/mL had moduli values of 147.67 kPa, which promoted myoblast differentiation into skeletal myotubes [64]. These findings suggest that the structural and functional mechanical properties of the myothreads described in this study may promote the development of mature tissue constructs and enable the development of multi-scale tissue constructs [105,106,107,108]. Future studies will focus on characterizing the relationship between the intrinsic stiffnesses of fibrin-based myothreads and the cellular mechanisms that direct myoblast differentiation and tissue maturation. We will also investigate myothread remodeling in terms of scaffold compaction, new matrix synthesis, and changes in cell density as a function of time.

The results of these studies also highlight the fact that the myogenic capability of cell-loaded myothreads may serve as a multifunctional scaffold to investigate the biological and biochemical mechanisms of skeletal muscle-fiber development and tissue maturation towards functional contractility for our future studies investigating innervation. To the best of our knowledge, cell-loaded fibrin microthreads are the first scaffolds that utilize fibrin to provide both regenerative biochemical signaling cues and fiber-like architecture to facilitate myogenesis without the use of provisional, sacrificial materials. In this study, we showed that myoblast-cultured myothreads provide cellular microniches to enhance myotube formation and maturation. Previous studies showed that myoblast-seeded constructs co-cultured with primary neurites or stem cell-derived neuronal cells enhanced the rate of myotube maturation and functional muscle regeneration, using in vitro systems [33,109,110,111]. Similarly, pre-vascularized skeletal muscle constructs fabricated from coaxial bioprinting of decellularized skeletal-muscle-matrix inks containing primary human skeletal myoblasts and HUVECs demonstrated higher contractile force relative to non-vascularized skeletal muscle constructs in vitro [112]. As such, we hypothesize that cell-loaded myothreads may serve as a platform technology to strategically analyze complex approaches for muscle maturation by incorporating myogenic stem cells or peripheral motor neurons, to enhance myogenesis and promote neurite outgrowth and neuromuscular junctions with the myotubes. We also anticipate that endothelial cells and pericytes co-cultured in myothreads may promote functional tissue regeneration. Together, these findings will enable the use of future studies that will incorporate additional cell types into myothreads, to potentially enhance the rate of functional muscle recovery, and will enable us to use myothreads as an in vitro benchtop model system to study the mechanisms of myogenesis and to develop layer-by-layer composites for implantation.

## 5. Conclusions

We present a simple biofabrication method that utilizes 3D printing of myoblast-loaded fibrin myothreads to facilitate myoblast alignment and myotube formation, as well as the formation of structural muscle constituents with mechanical properties similar to native skeletal muscle. We showed that myoblast loading densities affect the rate of myotube formation as well as myothread stiffness, after 9 days of culture. We anticipate that our myothread biofabrication approach will enable us to strategically investigate the biophysical and biochemical signaling cues and the cellular mechanisms that enhance skeletal muscle regeneration and functional tissue recovery for the treatment of volumetric muscle loss.

## Figures and Tables

**Table 1:** Mechanical properties of cell-loaded myothreads. Acellular (T) and cell-loaded fibrin myothreads (MyoT) were uniaxially stretched until failure. Varied densities (C1, C2, C4) and days of culture (D0, D9) were analyzed for effects on ultimate tensile strength, elastic modulus, and strain to failure. Loads to failure and different thread diameters are provided. Data are presented as average ± standard deviation.

	T/C0/D0	T/C0/D9	MyoT/C1/D0	MyoT/C1/D9	MyoT/C2/D0	MyoT/C2/D9	MyoT/C4/D0	MyoT/C4/D9
**Ultimate Tensile Strength (kPa)**	533 ± 281	508 ± 416	449 ± 337	256 ± 94	352 ± 112	294 ± 154	403 ± 213	207 ± 103 *
**Elastic Modulus (kPa)**	411 ± 209	433 ± 275	378 ± 198	191 ± 99^†^	379 ± 237	188 ± 121 ^†^*	354 ± 164	170 ± 65 ^†^*
**Strain to Failure (mm/mm)**	1.30 ± 0.26	1.25 ± 0.39	1.43 ± 0.29	1.20 ± 0.23	1.32 ± 0.15	1.46 ± 0.50	1.41 ± 0.36	1.14 ± 0.52
**Load (mN)**	47.4 ± 21.7	45.4 ± 16.0	62.4 ± 25.5	45.1 ± 17.4	49.2 ± 14.8	36.7 ± 21.1	51.2 ± 22.8	34.0 ± 19.2
**Diameter (µm)**	419 ± 169	387 ± 83	431 ± 121	469 ± 111	397 ± 62	407 ± 106	458 ± 137	469 ± 98
**Sample Number**	**10**	**9**	**10**	**9**	**9**	**10**	**11**	**11**

* indicates significant difference with T/C0/D9. ^†^ indicates significant difference within same densities at different timepoints (MyoT).

## Data Availability

The original contributions presented in the study are included in the article; further inquiries can be directed to the corresponding author. Similarly, requests for computer codes used in data analyses should be directed to the corresponding author.
